# The Influence of Financial Incentives on Vaccination Hesitancy: A Narrative Review of Recent Research

**DOI:** 10.3390/vaccines13030256

**Published:** 2025-02-28

**Authors:** Jason Wong, Camrin Gill, Amir Abdo, Ava Eisa

**Affiliations:** College of Pharmacy, Western University of Health Sciences, 309 E. Second St., Pomona, CA 91766, USA; camrin.gill@westernu.edu (C.G.); amir.abdo@westernu.edu (A.A.); ava.eisa@westernu.edu (A.E.)

**Keywords:** vaccine, hesitancy, monetary, financial, incentive

## Abstract

Background: Vaccine hesitancy represents a significant global health challenge that greatly hinders public health efforts focused on managing the transmission of infectious diseases. A wealth of original research conducted worldwide has examined various incentives that could help alleviate vaccine hesitancy and increase vaccination rates. Although some findings are conflicting, no comprehensive review has yet assessed the overall effectiveness of these strategies. This study aims to bridge this knowledge gap by examining how financial incentives influence people’s willingness to undergo vaccination. Methods: In August 2024, we extensively searched four databases for studies focusing on financial incentives and vaccination rates. Examples of financial incentives included lottery tickets and hypothetical or physical monetary rewards ranging in various amounts depending on the study. We selected nineteen relevant articles from a larger pool and evaluated them for validity and bias. Results: Around eighty percent of the research focused on COVID-19 vaccines, driven by the ongoing pandemic and the debates surrounding their use. Most of the studies indicated a positive influence of financial incentives on vaccination rates, although they often came with a higher risk of bias. Conversely, several studies suggest that financial incentives do not result in benefits. Instead, they highlight other factors that have a more profound effect on influencing people to undergo vaccination. The remaining studies are inconclusive regarding the effectiveness of financial incentives, concluding the need for further research. The strategies to mitigate these concerns included a combination of legal and monetary incentives. Summary: The effectiveness of financial incentives in boosting vaccination rates seems to differ significantly based on the region and context. They tend to be more effective in economically disadvantaged developing countries. In contrast, in developed nations, they may be ineffective or counterproductive due to various confounding factors such as financial background, lack of trust in the healthcare system, and/or lack of patient education. In resource-rich areas, educational programs often yield better results, and addressing widespread mistrust in healthcare systems and governmental policies through transparency is essential. Ultimately, employing tailored incentives alongside public education could enhance vaccination acceptance, particularly in culturally diverse countries like the United States, where understanding community preferences is crucial.

## 1. Introduction

The term “vaccine hesitancy” refers to the unwillingness or refusal to receive vaccinations, even when vaccination services are accessible [1]. This hesitancy poses a major challenge to public health, particularly to efforts to control the spread of infectious diseases. It is essential to understand that vaccine hesitancy may have many causes. It can stem from a complex interplay of personal beliefs, cultural influences, socio-demographic factors, misinformation, and other external motivators [1,2]. The World Health Organization (WHO) identifies vaccine hesitancy as one of the most critical threats to global health [3].

The introduction of new vaccines, including COVID-19 vaccines, has highlighted the need to understand the factors that drive vaccine hesitancy among adults. Reducing vaccine hesitancy is essential for achieving herd immunity and safeguarding individuals who cannot be vaccinated because of age or medical conditions [3]. Beyond preventing the spread of communicable diseases, addressing vaccine hesitancy can reduce healthcare costs associated with outbreaks and contribute to overall public health and safety. Healthcare systems employ various strategies to combat hesitancy, such as adequate education, enhancing accessibility, and implementing incentives [4].

Research has shown mixed evidence about the effectiveness of monetary incentives in boosting vaccination rates. In a survey involving over 300 U.S. adults, a hypothetical financial incentive of USD 600 increased the willingness to receive a vaccine by more than 25%, while a USD 1200 incentive raised that willingness to over 30% [5]. However, other research suggests that monetary incentives may not be as effective in increasing vaccination rates as alternative strategies, such as legal incentives. For example, a study in Italy found that implementing vaccination health certificates—needed for entry to certain venues and travel—significantly boosted vaccination rates [6].

This study investigates various aspects of vaccine hesitancy, particularly emphasizing how financial incentives may affect individuals’ willingness to receive vaccinations. There exists a knowledge gap regarding the efficacy of such incentives in reducing vaccine hesitancy, and no comprehensive research has yet evaluated their overall impact. While some studies suggest that incentives are effective, others argue the opposite, and a synthesis of these findings is lacking. Our research aims to provide insights into this knowledge gap to benefit public health campaigns and vaccination initiatives by assessing the impact of financial incentives from previous studies.

## 2. Materials and Methods

In August 2024, we conducted a comprehensive search across four databases—PubMed, Embase, Science Direct, and Web of Science—to identify articles examining the impact of incentives on vaccination rates. This investigation was prompted by our need to understand how various strategies can enhance vaccine uptake, particularly regarding financial incentives. Our literature search included keywords “vaccine”, “hesitancy”, “monetary”, “financial”, and “incentive” to identify relevant studies addressing our research question. We focused on studies published within the last ten years, from 2014 to 2024, to ensure our findings were based on the most up-to-date data and insights. This ten-year span was selected to broaden the types of vaccines that have been researched in recent years regarding the effect of monetary incentives on those who are vaccine-hesitant. The goal of the timeframe selection was to keep this paper up to date by only utilizing recent years’ research; however, we did not want to limit the research to only five years, as this filter caused a high yield of COVID-19-specific results. We believe that the COVID-19 pandemic significantly affected the landscape of vaccine hesitancy, and we wanted to see the pattern of the impact of monetary incentives over the years with additional types of vaccines. The purpose of using the ten-year span was to make our research generalizable to various vaccines. Additionally, we chose to implement an English filter on all of our searches to be able to accurately and critically appraise and evaluate the articles by using a common language between all authors.

Additionally, we set inclusion and exclusion criteria when selecting publications to include in our research to standardize the research. Articles were included in our research paper if they assessed the direct relationship between monetary incentives and vaccine uptake, if they were published in the last ten years, and if they were original research studies. Furthermore, articles were excluded if they were systematic reviews published prior to 2014 that assessed caregivers’ willingness to vaccinate infants instead of one’s willingness to undergo vaccination themselves and if they did not assess the impact of monetary incentives directly on vaccine hesitancy.

We began our research with PubMed using the previously mentioned keywords and filters. These keywords and filters produced 30 articles in PubMed, and 13 articles were chosen for further review. Out of the seventeen excluded articles, five were excluded because they were systematic reviews, eleven were excluded because they did not assess the relationship between monetary incentives and vaccine uptake, and one was excluded because it discussed a caregiver’s willingness to vaccinate infants.

Our research continued by using Embase. In this database, we used the same search terms as previously mentioned. Although we attempted to conduct a ten-year search, Embase did not have any relevant articles past 2021, which limited our search period to 2021–2024. This search resulted in 14 articles, but only 1 article met our inclusion criteria. The other 13 were excluded because 4 were systematic reviews and were therefore not original research articles, 3 did not assess monetary incentives to address vaccine hesitancy, and 6 were repeat studies that we found from the PubMed search.

The next database we searched was Science Direct, in which we utilized similar search terms. When we attempted to set a filter for publications from 2014 to 2024, we found that Science Direct had no relevant articles for 2014 or 2017. Therefore, research using Science Direct had two years of missing data. A large pool of 102 results came from this search, from which we ultimately selected only 5 articles that aligned with our focus. Therefore, 97 articles were not included, as they met various exclusion criteria. In total, 25 articles were excluded because they were systematic reviews of multiple research papers, 69 were excluded because they did not contain the keywords and were not relevant to the assessment of the effect of monetary incentives on vaccine hesitancy, and 3 were excluded because they were repeat studies from PubMed and/or Embase.

Lastly, we used the same key terms to search the Web of Science. We attempted to set a ten-year filter again but found no relevant articles past 2020 on Web of Science, limiting our research to the past four years. This search provided 11 results, of which none were included in the study. They were excluded because 4 did not assess the effectiveness of monetary incentives on vaccine hesitancy, 1 was a systematic review, and 6 were repeat studies from PubMed, Embase, and/or Science Direct.

**Figure 1:**
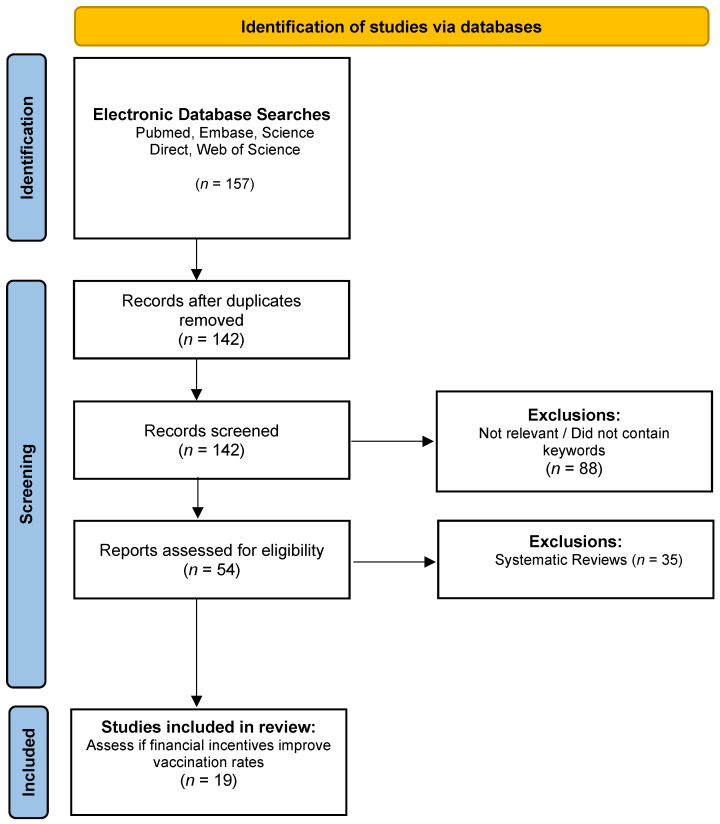
PRISMA flow diagram summarizing the literature search screening process.

The appraisal process involved various tools tailored to the specific type of study conducted. For randomized controlled trials, we utilized the Revised Cochrane Risk-of-Bias Tool for Randomized Trials (RoB 2). In contrast, we applied the Appraisal Tool for Cross-Sectional Studies (AXIS Tool) for cross-sectional and survey studies. Lastly, we utilized the Newcastle–Ottawa Scale (NOS) to appraise the case study. Our goal was to ensure the integrity and reliability of our findings as we examined the effectiveness of incentives in addressing vaccine hesitancy. In analyzing these selected studies, we aimed to provide meaningful insights that can enhance public health strategies and vaccination efforts. Refer to Figure 1 for the PRISMA flow diagram summarizing the literature search screening process. In this chart, the total number of studies found through all the different databases were combined, and this illustrates why some studies were excluded from the research. Most of the studies were excluded due to their not being relevant to the specific study topic and/or being a systematic review rather than an individual study. This led to a final count of 19 studies to be reviewed and included in this research paper.

## 3. Results

In this study, we appraised and included nineteen pertinent articles deemed essential for evaluating the relationship between incentives and vaccine hesitancy. This selection process stemmed from a wide-ranging search to gather diverse insights on the topic. For a clearer understanding of the included studies, their study periods, bias risks, study locations, and study types, a detailed overview is presented in Table 1.

### 3.1. Research That Shows Monetary Incentives Improve Vaccination Hesitancy

Monetary incentives have proven effective in improving vaccine hesitancy in developing countries, while other factors were involved in developed nations such as Germany. Combining incentives with restrictive laws for unvaccinated individuals in Germany has significantly increased the vaccination rate. In both developed and developing countries, studies have shown that older adults were more likely to receive vaccines when incentives were offered, suggesting the vulnerability of this demographic to financial hardship and their likelihood of responding positively to such measures. In the United States, responses to incentives and vaccination have varied widely, reflecting the country’s diverse cultural and socio-economic composition.

Using monetary incentives, including cash and non-financial incentives like food vouchers and gifts, positively influenced COVID vaccination rates and other immunization uptake. This was highlighted in a 2021 study conducted by Afsharinia et al. [7], which involved 1131 Indian adults and utilized a telephonic questionnaire. The findings indicated that vaccination acceptance significantly improved with the implementation of incentives. While the study demonstrated a clear correlation between offering monetary incentives and increased readiness to receive a vaccine, individual responses may vary based on the source of vaccine-related information and incentives. The Indian participants generally exhibited greater trust in leaders from healthcare, political, and social sectors whom they previously recognized as reliable sources.

In a study that was conducted by Fishman et al. [12], on the effectiveness of monetary incentives—specifically USD 1000, USD 200, and USD 100 cash payments—and mandatory COVID-19 vaccination among 3698 unvaccinated individuals 50 years old and younger, predominantly female and U.S. residents, it was found that both interventions effectively increased vaccination rates. Additional measures were also implemented, such as a USD 1000 tax benefit for vaccinated individuals and a USD 1000 tax penalty for the unvaccinated. Notably, the USD 1000 guaranteed cash incentive proved the most effective, increasing willingness to vaccinate by 17.1%. The other cash incentives, of USD 200 and USD 100, also positively impacted vaccination willingness, by 9.2% and 9.3%, respectively. The USD 1000 tax penalty for unvaccinated individuals emerged as the second most effective strategy, contributing to a 13.8% increase in vaccination rates. Finally, the employer mandate significantly influenced vaccination rates, resulting in an 8.6% increase, compared to just 1.4% for other mandates, such as those requiring vaccination for entry into restaurants or public places.

The survey conducted by Andresen et al. in 2022 [5] assessed the impact of hypothetical incentives of USD 600 and USD 1200 on the vaccination intentions of 346 vaccine-hesitant adults in the US regarding COVID-19. The results revealed that 26.89% of the participants were willing to receive the vaccine with a USD 600 incentive, while 30.06% were willing with a USD 1200 incentive. These findings suggest that financial incentives may effectively increase vaccination rates, irrespective of the reasons why individuals hesitate over receiving the COVID-19 vaccine.

In Sweden, a survey conducted by Campos-Mercade et al., in 2021 [10], revealed that monetary incentives boosted vaccination. In total, 8286 participants were asked whether payments could increase their likelihood of receiving a vaccine, and the outcome revealed that offering a USD 24 (or SEK 200) incentive resulted in a 4.2% increase in the vaccination rate. This finding underscores the potential of incentives and highlights the effect of incentives on vaccination rates, even in nations with an increased vaccination rate, such as Sweden, which has a baseline vaccination rate of 71.6%. Other strategies, such as behavioral nudges, were studied but did not significantly improve individuals’ vaccination intentions. The findings from this study demonstrate how small monetary incentives can raise immunization rates.

In 2022, Sprengholz et al. [18] conducted a cross-sectional study that revealed that both financial incentives and legal mandates significantly increased the COVID-19 vaccination willingness among participants in Germany. Financial incentives encouraged individuals to reconsider their stance on vaccination by providing compelling reasons to undergo vaccination. Simultaneously, legal requirements, such as restrictions imposed on the unvaccinated, also effectively influenced individuals’ willingness to receive the vaccine. Many participants indicated that the threat of facing limitations or penalties was a great motivation for them to undergo vaccination. Overall, the findings underscore the effectiveness of combining monetary and legal strategies to enhance public health initiatives during a pandemic.

Financial incentives have demonstrated their effectiveness, particularly in developing countries and among vulnerable populations. In a study conducted by Yue et al., in 2020 [22], the effects of financial incentives were evaluated among individuals aged 65 and older in Singapore to assess whether these incentives would impact the influenza vaccination rate. The study involved 4000 participants divided into four groups, each receiving different shopping voucher incentives for completing a questionnaire and obtaining a vaccination at their own expense. The results revealed that a minor increase in the reward’s size from SGD 10 to 20 boosted vaccination participation by 3%. Furthermore, raising the incentive to SGD 30 led to an increase in participation to 9.2%. Notably, non-working elderly individuals responded more positively to changes in incentives, with the effectiveness varying by social class, race, and family size but not significantly differing by educational level, gender, or age. The findings indicate that incentives ranging from SGD 10 to 20 can effectively enhance vaccination rates while keeping costs manageable for health systems.

In the study by Bonner et al. (2023) [9], researchers administered a discrete survey experiment to adults in Minnesota to investigate the motivations behind their influenza vaccine acceptance. The study focused on various factors, including financial incentives, ease of vaccine access, and effective communication through messaging and informational sources. The findings indicated that financial incentives significantly increased participants’ willingness to vaccinate. Moreover, simplifying access to vaccination—through convenient locations and reduced wait times—proved crucial in encouraging vaccine uptake. Effective messaging, which underscored personal and community benefits, was key in fostering positive attitudes toward vaccination. Additionally, trust in information sources, especially healthcare providers and public health organizations, was essential in influencing vaccination decisions. Overall, the results emphasize that combining these factors enhanced influenza vaccine uptake among adults, with ease of access emerging as the most impactful element, surpassing the effect of financial incentives.

A randomized controlled trial conducted by Shen et al. in 2024 [16] demonstrated that financial rewards effectively boosted flu vaccination rates in a group of older adults in China. The study revealed that the participants who received financial incentives were more likely to receive a vaccine than those who did not. This trial highlights financial incentives as a promising strategy to enhance vaccination uptake in older populations, potentially bolstering public health initiatives against influenza. While the findings indicate that financial incentives can effectively boost short-term vaccination rates and expedite the immunization process in China, they also suggest that no single financial incentive results in long-term changes in future vaccination behaviors or the establishment of consistent vaccination habits.

Barber et al. [8] investigated the impact of incentives through the Conditional Cash Lottery (CCL) program, introduced to two distinct age groups in Ohio. Conducted by the Ohio Department of Health from May to June 2021, the initiative encouraged individuals to receive an initial COVID-19 shot and to participate in the CCL program. One adult (aged over 18) could win USD 1 million each week, while one youth (aged 12–17) could win a full college scholarship. As a result, there was a noticeable improvement in vaccination rates, beginning with the program’s launch. In the first week alone, the vaccination rate increased by 0.31 percentage points and eventually rose to 0.6 percentage points. By the program’s conclusion, in June 2021, there was an overall increase of 1.5% in COVID-19 vaccinations. The studies highlighting the positive impact of monetary incentives on addressing vaccine hesitancy are presented in detail in Table 2.

### 3.2. Research That Shows Monetary Incentives Fail to Change Attitudes or Behaviors Related to Vaccine Uptake

In several studies, monetary incentives have not effectively increased individuals’ willingness to be vaccinated. Research findings indicate that vaccine hesitancy is a complex issue shaped by numerous factors, such as misinformation, distrust, and cultural beliefs. These elements significantly shape individuals’ attitudes toward vaccines. Although monetary or legal incentives were employed to address these barriers, the results varied. The cross-sectional survey by Bennett et al. (2022) [2] found that monetary incentives did not notably increase the COVID-19 vaccination rate. Instead, the most effective motivators for participants were “increased insurance costs” and the rise of “more transmissible and dangerous variants”.

A cross-sectional study conducted by Barello et al. in 2023 [6] revealed that the legal incentives implemented by Italy during the COVID-19 pandemic—specifically, the implementation of vaccination health certificates that are required for accessing certain venues and for travel—proved to be more effective in motivating booster vaccinations than monetary incentives. The restrictions preventing individuals from attending public events or traveling without proper documentation encouraged higher vaccination uptake than tax and fee incentives. This study offers valuable insights for policymakers seeking to enhance vaccination rates and address vaccine hesitancy in an ongoing pandemic.

A survey by Shmueli, published in 2022 [17], among 461 adults in Israel between December 2020 and January 2021, soon after the COVID-19 vaccine was made available, revealed that monetary incentives and the provision of vaccination certificates for entry to public venues such as hotels and restaurants did not enhance participants’ motivation or urgency to receive the vaccine.

Chang et al. (2023) [11] conducted a substantial preregistered randomized controlled trial that involved 57,893 individuals in Northern California. The study aimed to evaluate the effects of personalized reminder messages and a modest financial incentive of USD 25 on the rates of COVID-19 booster vaccinations. The results revealed that personal reminder messages significantly increased vaccination rates within two weeks, demonstrating their effectiveness in motivating individuals to schedule and receive booster shots. In contrast, the small financial incentive did not lead to any additional improvements in vaccination rates, indicating that monetary rewards were ineffective in this context. Overall, the findings highlight the potential of low-cost, targeted reminder strategies to enhance booster vaccination uptake, suggesting that financial incentives may not be necessary to achieve desired public health outcomes.

A study conducted by Gong et al. in 2023 [13], which utilized a 38-item questionnaire distributed to outpatient hospitals in Cleveland, Ohio, found that monetary incentives did not lead to higher vaccination rates. Only 0.95% of participants reported that the Ohio Vax-a-Million lottery made them more likely to undergo vaccination, while 29.7% felt it reduced their likelihood of being vaccinated. Additionally, 6.8% reported that the USD 100 incentive increased their likelihood of undergoing vaccination, whereas 17.4% believed it would decrease their likelihood. Moreover, 20.6% stated that news of the delta variant heightened their likelihood of being vaccinated. Overall, this survey suggested that monetary incentives did not lead to higher COVID-19 vaccination rates; in fact, a larger number of participants felt that these incentives reduced their willingness to be vaccinated.

It is important to note that some studies that showed no benefit in using monetary incentives to improve vaccine hesitancy revealed several issues. The first was ethical concerns. Paying someone to be vaccinated can seem like coercion and may not reflect a person’s genuine willingness to be vaccinated. It conceals the participant’s reasons for not wanting to be vaccinated and does not address the real concerns that the individual has about receiving vaccines. Additionally, this can cause unintended consequences, as these participants will be likely to expect financial compensation for future vaccinations. Many of the studies also noted that the participants became even more hesitant over receiving a vaccine when offered financial incentives in return, as this made them feel like something was wrong with the vaccine. Incentivizing vaccinations can lead to a further lack in the healthcare system and could cause individuals to become more hesitant. It is also not ethically appropriate that only people who are hesitant over receiving vaccines should receive an incentive. The studies that did not show that monetary incentives benefit vaccine hesitancy are summarized in Table 3.

### 3.3. Research That Showed Monetary Incentives May Have Varying Effects on Vaccine Hesitancy and May Not Always Lead to a Reduction in Concerns

In several studies, the provision of incentives showed little impact on participant behavior. For instance, the survey by Ostermann et al. (2023) [14] asked 406 pregnant women in Tanzania and Sub-Saharan Africa about their willingness to vaccinate their children under two scenarios: (1) receiving an incentive and (2) facing a vaccination cost. The study revealed that these women were not motivated by monetary incentives. Instead, they preferred non-monetary options such as mobile phone credit, pharmacy vouchers, lottery tickets with a high chance of winning, birth certificates, and free maternal health checks. Additionally, about 97% of the women expressed a willingness to pay for timely vaccinations for their children.

In their study, Sprengholz et al., in 2022 [19], examined data from Germany involving 2701 subjects to identify factors influencing primary and booster COVID-19 vaccinations and the effects of integrating vaccination mandates with financial incentives. The results indicated that vaccinated individuals had a clearer understanding of the benefits of vaccination and were less likely to believe in conspiracy theories. On the other hand, unvaccinated individuals tended to disregard both vaccination and mandates. However, introducing monetary incentives (up to EUR 2000) significantly increased the willingness to be vaccinated across all three groups, particularly in the absence of mandates. The study concluded that financial incentives were unlikely to convince unvaccinated individuals but could significantly increase booster uptake in health policies. The results indicated that reasonable incentives should be provided to achieve this goal.

A cross-sectional study conducted in Austria by Stamm et al., in 2022 [20], found that 70% of unvaccinated individuals reported no motivation to receive the COVID vaccine. However, their willingness to be vaccinated increased when incentives were offered, such as free vaccines, a vaccination lottery, and a monetary reward of EUR 100. These different incentives made an impact, showing that extrinsic and financial incentives may work, but that they have no behavioral effect on society. Over time, this may “crowd out” individuals’ intrinsic motivators.

A survey conducted by Taber et al. in 2023 [21] found that about 42% of participants were unwilling to accept any lottery-based monetary incentives for vaccination. The participants were given a list of 12 lottery structures and asked to indicate their preferences. On average, they favored options that awarded smaller amounts of money to more people, rather than larger sums to fewer individuals. This demonstrates that different lottery structures might have similarly (un)motivating effects on unvaccinated adults.

Conversely, a survey conducted by Raman et al. in 2022 [15], among 548 previously vaccinated Americans, revealed an increased willingness to receive a COVID-19 booster when presented with higher monetary incentives, paid days off work, and considerations regarding the vaccine manufacturer and efficacy. Incentives did enhance the willingness to receive a COVID-19 booster; however, the ranking of the incentives did not suggest that all monetary rewards are as impactful at boosting vaccination rates, as individuals ranked a pay day off work higher than the USD 10 monetary incentives. This shows how different incentives can help increase individuals’ willingness to receive a vaccination, not necessarily monetary incentives. The studies that showed no results regarding the benefits of monetary incentives regarding vaccine hesitancy are summarized in Table 4.

Our results show that monetary incentives may be impactful for those who are vaccine-hesitant, even in areas where there is high vaccine uptake. The main issues behind monetary incentives not motivating people to be vaccinated were further distrust in the healthcare system, since the participants were being paid or because they had other concerns that monetary incentives could not overcome, such as social beliefs and lack of vaccine education. As mentioned earlier, incentivizing people to receive vaccinations can lead to many ethical concerns. Our findings show that although financial incentives can improve vaccine hesitancy, understanding the underlying causes of people’s hesitancy is the key to addressing this issue.

## 4. Discussion

### 4.1. Incentives and Global Vaccine Decisions

Numerous studies have explored the role of incentives in enhancing vaccination uptake globally. While it is challenging to attribute increased vaccination rates solely to monetary incentives, their impact appears more pronounced in developing nations. As noted by Afsharinia et al. (2021) [7], Indian adults facing economic hardship are more likely to receive vaccinations when offered small, guaranteed financial incentives to help cover related costs such as transportation, childcare, or lost wages. Similarly, Yue et al. (2018) [22] found that providing shopping vouchers as monetary incentives significantly boosted influenza vaccination rates among the non-working elderly population in Singapore. Often facing financial difficulties, this demographic represents some of society’s most vulnerable and at-risk members. Thus, financial incentives not only serve as effective motivational tools for the elderly to be vaccinated, but also contribute to increasing vaccination rates in a context in which the influenza vaccine entails a copayment.

While monetary incentives are often perceived as more effective in developing countries, this pattern can vary depending on the context and target population. Some studies have shown that participants prioritize non-financial incentives over monetary ones. For instance, in Tanzania, pregnant women favored non-monetary incentives and were willing to pay to guarantee that their children would receive routine vaccinations in a timely manner [14]. Non-financial benefits, such as obtaining a birth document and free routine checkups for mothers, were considered more valuable [14]. These incentives often carry higher costs and may be less accessible, influenced by family dynamics and how funds are allocated within certain communities [14]. This suggests that monetary incentives are not always the sole factor influencing an individual’s decision to accept vaccinations. Instead, other factors, shaped by regional values and priorities, may also play a significant role.

In contrast, developed countries showed less interest in financial incentives and tended to prioritize other types of benefits. An Italian study, by Barello, highlighted that participants valued legal incentives, like COVID-19 vaccine certificates and travel privileges, more highly than monetary incentives [6]. The participants ranked options such as tax relief and fees for being unvaccinated above cash rewards [6]. This suggests that offering monetary incentives can lead to mixed reactions in developed regions. As Shmueli noted in a survey of the Israeli population, paying individuals to undergo vaccination might raise doubts about the necessity of taking personal responsibility for health [17]. Many feel that financial rewards do not align with societal values emphasizing hard work to protect health. Additionally, monetary incentives could cause people to question the true intentions behind vaccine promotion [17]. In these societies, financial rewards are sensitive; small payments often fail to motivate individuals, while larger sums, although potentially effective, may not be practical or manageable for governments.

The role of incentives has been examined in various studies, revealing notable differences in responses across global populations. The findings were remarkably diverse in the United States, which is to be expected, given the country’s mix of individuals from different backgrounds. For example, Andresen discovered that monetary incentives positively influenced acceptance of the COVID-19 vaccination [5]. U.S. adults uncertain about being vaccinated were more inclined to undergo vaccination when offered financial rewards, especially compared to outright refusing the vaccine [5]. As a result, the willingness to be vaccinated increased from 25% to 33% when a higher monetary incentive was introduced [5]. Similarly, Bonner reported that 80% of participants in Minnesota were willing to be vaccinated when vaccine sites were accessible, noting that financial incentives significantly impacted flu vaccine acceptance among younger participants aged 18 to 40 [9].

In various studies, monetary incentives did not effectively increase vaccination rates among U.S. residents. Factors such as race, insurance coverage, and socioeconomic status must be considered due to the diversity within the population [4]. In Spees’ research on obstacles to COVID-19 vaccination, the most frequently reported issue was a lack of information and negative attitudes toward the vaccine [4]. Notably, the informational gap was primarily noted among the White participants, in contrast to the Black and Latino groups [4]. Addressing these barriers is crucial, especially within a racially and culturally diverse U.S. population. Given that monetary incentives elicit varied responses, focusing on education may lead to a more consistent reaction across different communities.

Key reasons for vaccine hesitancy that affect both developed and developing nations are widespread information insufficiency and lack of confidence in entities such as healthcare and governmental institutions. As Bennett highlighted, vaccine acceptance tends to increase with age and higher education levels among participants in the U.S. [2]. In contrast, those who questioned vaccine effectiveness often expressed skepticism about the motives of the government and healthcare providers [2]. Furthermore, vaccine hesitancy was notably more prevalent among individuals who believed their health was dictated by divine intervention, reflecting a gap in clinical knowledge and trust in medical systems. This mistrust in government is not exclusive to the U.S.; it has been observed globally, including in countries like Austria. Stamm’s survey revealed that participants lacked trust in societal and political institutions, particularly the government [20]. Although confidence in the media and pharmaceutical companies was also low, individuals tended to place greater trust in the healthcare system and scientific research [20].

While monetary incentives can be an effective strategy, particularly during urgent public health crises like pandemics, to boost vaccination rates and achieve herd immunity—thus protecting vulnerable individuals, especially the immunocompromised—they may only offer a temporary solution. Relying on financial incentives could set a precedent for future healthcare interventions, making them contingent on monetary rewards. We need to implement systemic and sustainable strategies to tackle vaccine hesitancy effectively. This includes building trust in healthcare systems, ensuring transparency regarding vaccine safety, and enhancing public education. Trusted healthcare providers and community leaders should communicate openly, using targeted advertising on reputable social media platforms to counteract misinformation and reduce distrust. On a more localized level, initiatives such as incorporating vaccine education into school curricula and training healthcare providers to communicate more effectively with patients can help to identify and address personal barriers, ultimately fostering social change and helping to eliminate vaccine hesitancy.

In conclusion, establishing a clear framework is essential for guiding future research and improving clinical practices. This involves creating effective methods to measure vaccine hesitancy, enabling us to tackle potential barriers more effectively. As highlighted in the study by Barello, policymakers should consider implementing non-monetary strategies that align with societal values and priorities [6]. For instance, measures such as mandating vaccine certificates for entry to public spaces, workplaces, and events could be explored. These strategies may enhance compliance while addressing vaccine hesitancy through targeted educational campaigns designed to address vaccine hesitancy, promote understanding, and encourage acceptance within the population. Understanding the underlying causes of vaccine hesitancy across different cultural and regional contexts necessitates longitudinal studies evaluating which interventions are genuinely effective and most suited to each culture. Conducting community-based research can reveal the motivations behind vaccine hesitancy and identify appropriate interventions for various populations. Strategies such as incorporating questionnaires into routine patient visits at healthcare facilities, integrating vaccinations into standard care practices, and assessing the effectiveness of these interventions can significantly contribute to this effort.

### 4.2. Limitations

This study aimed to provide a thorough and impartial literature review on how financial incentives affect vaccination acceptance. However, the search did not include non-English publications, which may have excluded some pertinent studies. There could have been more viable and robust research regarding the use of monetary incentives for vaccine-hesitant populations in other languages. Moreover, Embase and Web of Science had no relevant articles past 2021 and 2020, respectively. This limits the data, as most of the studies from 2020 onward primarily focus only on COVID-19 vaccines, and the data are therefore not translatable to other common vaccines. Additionally, it is worth noting that the COVID-19 vaccines were authorized by the FDA for emergency use during these studies, rather than receiving full approval, which might also have influenced the outcomes of the selected studies. It is important to note that the study selections were inconsistent in all the articles. Some articles focused primarily on patients who had not yet received vaccinations, while other studies used random sample groups to gather their data. This may have led to selection bias and possible contamination of the results. All of these limitations may impact the generalizability of the findings, since the research yielded specific filters, gaps in years of research, and various types of bias.

## 5. Conclusions

The effects of financial incentives on individuals’ willingness to receive vaccinations can vary significantly. While some people may be encouraged by cash rewards or other monetary benefits to overcome their hesitations, others might remain unconvinced by such offers, suggesting the multifaceted nature of vaccine hesitancy, which extends beyond financial motivations. As a result, socioeconomic, cultural, and psychological factors must be considered when assessing vaccine hesitancy.

After reviewing multiple original studies from 2014 to 2024, it is evident that the influence of financial incentives on vaccination rates varies significantly by region and context. Our analysis is based on 19 publications, which makes it challenging to draw definitive conclusions about the global impact of monetary incentives on vaccine acceptance. While financial incentives may seem more effective in developing countries where populations face economic hardships, this view does not capture vaccine hesitancy complexities. Factors such as cultural norms, economic status, demographic variations, individual perceptions of health systems, and the level of trust in health institutions play crucial roles in this issue. Conversely, in developed countries, these incentives often prove ineffective and can sometimes backfire, raising concerns about vaccine safety or government motives. In more affluent regions, educational programs are likely to be more effective than financial incentives in encouraging individuals to receive vaccinations. Additionally, a prevalent issue around the globe is the widespread mistrust of healthcare systems and government policies. For this reason, offering financial incentives to increase vaccinations may worsen this mistrust. For this challenge to be addressed, the root cause needs to be identified through future studies, and policies must be implemented and monitored.

The effectiveness of financial incentives largely depends on various social and regional factors. In countries where trust in institutions is low or cultural barriers exist, monetary incentives alone may not effectively curb vaccine hesitancy. Instead, a tailored approach that combines customized incentives with public education could lead to higher vaccination rates and greater acceptance. For instance, in urban populations, fostering partnerships between healthcare providers, policymakers, local organizations, community leaders, and trusted influencers can facilitate the spread of accurate and culturally appropriate information. Integrating monetary incentives into vaccination programs promoted by these trusted entities can enhance public acceptance. As noted in Table 2, Afsharinia highlighted the positive impact of trusted sources on the Indian population’s vaccination readiness. These programs should also be continuously monitored and evaluated to ensure effectiveness and optimize outcomes. In contrast, rural populations with limited access to healthcare and information may benefit from alternative approaches, such as mobile vaccination clinics, culturally suitable education programs, and context-appropriate incentives, which can build trust and improve vaccination rates. As mentioned in the study by Ostermann et al., monetary incentives were not the primary motivators for the women surveyed in Southern Tanzania [14]. Instead, they showed a preference for non-monetary rewards, which highlights that the individuals’ preferences for incentives were shaped by cultural and contextual concepts, emphasizing the need for tailored approaches that align with the needs and values of the targeted population. In culturally diverse countries like the United States, healthcare and social organizations can significantly benefit from conducting polls at both state and national levels as part of future research. This approach would help to identify the most appealing incentives for the population and guide the effective implementation of vaccine incentivization strategies.

## Figures and Tables

**Table 1:** Overview of all the reviewed studies used in the study.

Study	Article	Study Time Period	Location	Type of Study	Appraisal Tool	Risk of Bias
Afsharinia et al., 2023 [7]	Role of leadership and incentive-based programs in addressing vaccine hesitancy in India	2021	India	Cross-sectional quantitative study	AXIS Tool	Moderate
Andresen et al., 2022 [5]	The impact of financial incentives on COVID-19 vaccination intention among a sample of U.S. adults	2021	US	Survey	AXIS Tool	Moderate–High
Barber et al., 2022 [8]	Conditional cash lotteries increase COVID-19 vaccination rates	2021	US	Case study	NOS	Low
Barello et al., 2023 [6]	Providing freedom or financial remuneration? A cross-sectional study on the role of monetary and legal incentives on COVID-19 further booster vaccination intention in the Italian context.	2022	Italy	Cross-sectional study	AXIS Tool	Low
Bennett et al., 2022 [2]	Factors underlying COVID-19 vaccine and booster hesitancy and refusal, and incentivizing vaccine adoption	2021	US	Cross-sectional study	AXIS Tool	Low
Bonner et al., 2023 [9]	What motivates adults to accept the influenza vaccine? An assessment of incentives, ease of access, messaging, and sources of information using a discrete choice experiment	2019	US	Survey	AXIS Tool	Moderate
Campos-Mercade et al., 2021 [10]	Monetary incentives increase COVID-19 vaccinations	2021	Sweden	Randomized controlled trial	RoB2 Tool	Moderate–High
Chang et al., 2023 [11]	Reminders, but not monetary incentives, increase COVID-19 booster uptake	2022	US	Randomized controlled trial	RoB2 Tool	Low– Moderate
Fishman et al., 2022 [12]	Comparative effectiveness of mandates and financial policies targeting COVID-19 vaccine hesitancy: A randomized, controlled survey experiment	2021	US	Randomized, controlled, survey-embedded experiment	AXIS Tool	Low
Gong et al., 2023 [13]	Financial Incentives Are Associated with Lower Likelihood of COVID-19 Vaccination in Northeast Ohio	2021–2022	US	Survey	AXIS Tool	Low
Ostermann et al., 2023 [14]	Is the intention to vaccinate enough? Systematic variation in the value of timely vaccinations and preferences for monetary vs. non-monetary incentives among pregnant women in southern Tanzania	2017	Tanzania	Survey	AXIS Tool	Moderate
Raman et al., 2022 [15]	COVID-19 booster uptake among US adults: Assessing the impact of vaccine attributes, incentives, and context in a choice-based experiment	2021	US	Survey	AXIS Tool	Low– moderate
Shen et al., 2024 [16]	Effectiveness of financial incentives on influenza vaccination among older adults in China: a randomized clinical trial	2021	China	Randomized controlled trial	RoB2 Tool	Moderate
Shmueli, 2022 [17]	The Role of Incentives in Deciding to Receive the Available COVID-19 Vaccine in Israel	2020–2021	Israel	Online survey	AXIS Tool	Low– Moderate
Sprengholz et al., 2022 [18]	Payments and freedoms: Effects of monetary and legal incentives on COVID-19 vaccination intentions in Germany	2021	Germany	Cross-sectional study	AXIS Tool	Moderate
Sprengholz et al., 2022 [19]	Different Intervention for COVID-19 Primary and Booster Vaccination? Effects of Psychological Factors and Health Policies on Vaccine Uptake	2022	Germany	Online survey	AXIS Tool	Low– Moderate
Stamm et al., 2022 [20]	Coronavirus vaccine hesitancy among unvaccinated Austrians: Assessing underlying motivations and the effectiveness of interventions based on a cross-sectional survey with two embedded conjoint experiments	2021	Austria	Cross-sectional study	AXIS Tool	Low– Moderate
Taber et al., 2023 [21]	Experimental Tests of Hypothetical Lottery Incentives on Unvaccinated Adults’ COVID-19 Vaccination Intentions	2021	US	Online survey	AXIS Tool	Moderate
Yue et al., 2020 [22]	Optimal Design of Population-Level Financial Incentives of Influenza Vaccination for the Elderly	2018	Singapore	Randomized controlled trial	RoB2 Tool	Moderate

**Table 2:** Summary of studies showing the positive impact of monetary incentives.

Study	Article	Objective	Study Subjects	Methods	Results
Afsharinia et al., 2023 [7]	Role of leadership and incentive-based programs in addressing vaccine hesitancy in India	To examine the impact of incentive-based programs to reduce vaccine hesitancy in India	1131 Indian adults aged 18 years or older	Participants were tracked from July to November 2021, with data collected through telephone interviews.	Vaccination acceptance significantly improved with financial incentives.
Andresen et al., 2022 [5]	The impact of financial incentives on COVID-19 vaccination intention among a sample of U.S. adults	To evaluate the effect of financial incentives on intention to receive the COVID-19 vaccination	346 US vaccine-hesitant adults	The survey asked the participants about their willingness to be vaccinated if offered a hypothetical incentive of USD 600 or USD 1200.	Results indicated that more individuals would receive the vaccine if offered a financial incentive.
Barber et al., 2022 [8]	Conditional cash lotteries increase COVID-19 vaccination rates	To assess the efficacy of conditional cash lottery incentives to increase COVID-19 vaccination rates	Adults 18 years or older in Ohio	Participants were split into a lottery group offered cash prizes and a control group.	Financial incentives, particularly conditional cash lotteries, increased vaccination rates in areas experiencing vaccine hesitancy.
Bonner et al., 2023 [9]	What motivates adults to accept influenza vaccine? An assessment of incentives, ease of access, messaging, and sources of information using a discrete choice experiment	To look at motivating factors for receiving the influenza vaccine	1803 adults 18 years of age and older at a state fair	A 25-question survey was administered to adults aged 18 and older in which they evaluated 16 parameters to explore how factors affected willingness to receive the influenza vaccination.	Financial incentives improve vaccination rates, but increasing accessibility increases vaccine willingness the most.
Campos-Mercade et al., 2021 [10]	Monetary incentives increase COVID-19 vaccinations	To evaluate the effects of payments on COVID-19 vaccination uptake	8286 participants in Sweden	Participants, aged between 18 and 49, were randomly allocated to one of five treatment conditions or to a control group	Monetary incentives increased vaccination rates, even in areas with previously high vaccination rates.
Fishman et al., 2022 [12]	Comparative effectiveness of mandates and financial policies targeting COVID-19 vaccine hesitancy: A randomized, controlled survey experiment	To evaluate the effects of vaccine mandate vs. financial incentive on vaccine uptake	3698 unvaccinated US residents	Different vaccine policies were implemented, to determine the most influential technique to improve vaccine rates.	Financial incentives increased the likelihood of receiving a vaccine. Employer mandates were more effective than other mandate policies.
Shen et al., 2024 [16]	Effectiveness of financial incentives on influenza vaccination among older adults in China: a randomized clinical trial	To examine the short- and long-term effectiveness of varying financial incentives on increasing vaccine uptake	720 Chinese adults aged 18 or older	Various financial incentives for receiving the influenza vaccination were distributed.	Financial incentives significantly boosted the intention to vaccinate.
Sprengholz et al., 2022 [18]	Payments and freedoms: Effects of monetary and legal incentives on COVID-19 vaccination intentions in Germany	To examine the impact of monetary and legal incentives on COVID-19 vaccination rates	782 German adults aged 18–74 years	782 individuals randomly allocated into two experimental conditions, either legal or no legal incentive, with financial incentives.	Monetary incentives may boost vaccination uptake slightly; however, their expensive implementation costs raise concerns about the effectiveness of this approach.
Yue et al., 2020 [22]	Optimal Design of Population-Level Financial Incentives of Influenza Vaccination for the Elderly	To assess financial incentive effectiveness on vaccination uptake in the elderly	4000 Singaporean adults aged 65 years or older	1000 participants were randomly assigned into four treatment groups and provided with a monetary incentive to participate.	Vaccination rates increased when offered financial incentives. The non-working elderly group showed greater sensitivity to incentive changes than those employed.

**Table 3:** Summary of studies showing no benefit of monetary incentives.

Study	Article	Objective	Study Subjects	Methods	Results
Barello et al., 2023 [6]	Providing freedom or financial remuneration? A cross-sectional study on the role of monetary and legal incentives on COVID-19 further booster vaccination intention in the Italian context	To analyze the roles of financial and legal incentives in increasing COVID booster vaccination rates	674 Italian adults	Participants responded to five questions regarding their willingness to receive additional booster shots if incentives were offered.	Legal incentives, especially the necessity of having a COVID-19 vaccine certificate for entry to certain venues and travel, proved to be more impactful in encouraging individuals to receive the booster vaccine compared to financial incentives.
Bennett et al., 2022 [2]	Factors underlying COVID-19 vaccine and booster hesitancy and refusal, and incentivizing vaccine adoption	To evaluate reluctance regarding COVID-19 vaccination and how to encourage vaccine completion using incentives	3497 US adults	An online survey via Qualtrics was performed to determine the most effective incentive to increase vaccination rates.	The two most effective incentives were the risk of higher insurance costs and the rise of a new COVID variant that poses a greater threat. In contrast, the least common reasons for not being vaccinated were cost or inconvenience, while the main reasons contributing to hesitation toward vaccines included a belief that the vaccine was inessential, distrust, and safety risks.
Chang et al., 2023 [11]	Reminders, but not monetary incentives, increase COVID-19 booster uptake	To evaluate monetary incentives vs. reminders to encourage individuals to receive their COVID-19 booster shots	57,893 participants in Northern California, U.S.	Participants were divided into two groups: one received email reminders and the other received SMS reminders. Some reminders included a USD 25 incentive for vaccination, while others did not.	The vaccination rate was reduced in the group that was sent both a reminder and a monetary incentive compared to those who received only a reminder. This suggests that the monetary incentives did not significantly enhance vaccine rates beyond what the reminders alone achieved.
Gong et al., 2023 [13]	Financial Incentives Are Associated with Lower Likelihood of COVID-19 Vaccination in Northeast Ohio	To estimate the efficacy of monetary rewards in increasing of COVID-19 vaccine uptake in northeastern Ohio	471 US participants in Cleveland Veteran Affairs Hospitals	A questionnaire with 38 inquiries was administered to patients to see how cash rewards may impact their decision-making regarding vaccinating.	Neither incentive influenced an individual’s decision to be vaccinated, and a higher percentage of participants believed that these incentives had a reverse effect, making them less likely to be vaccinated.
Shmueli, 2022 [17]	The Role of Incentives in Deciding to Receive the Available COVID-19 Vaccine in Israel	To determine the incentive’s role in the decision to receive COVID-19 vaccines	461 unvaccinated adults in Israel	Individuals completed an online survey to evaluate their willingness to be vaccinated immediately, within three months, or within a year.	Financial rewards or vaccination certificates did not change an individual’s mind as to whether to prioritize receiving the COVID vaccination.

**Table 4:** Studies showing no results for monetary incentives.

Study	Article	Objective	Study Subjects	Methods	Results
Ostermann et al., 2023 [14]	Is the intention to vaccinate enough? Systematic variation in the value of timely vaccinations and preferences for monetary vs. non-monetary incentives among pregnant women in southern Tanzania	To examine the preferences of pregnant women’s intention to be vaccinated using monetary and non-monetary incentives	406 African pregnant women who were in their third trimester	A survey was conducted with 406 women to assess their willingness to vaccinate their children under two scenarios: (1) receiving an incentive or (2) having a vaccination fee.	Women were most likely to have their newborns vaccinated based on the routine vaccination guides, even without incentives.
Raman et al., 2022 [15]	COVID-19 booster uptake among US adults: Assessing the impact of vaccine attributes, incentives, and context in a choice-based experiment	To assess the influence of incentives on COVID-19 vaccination	548 fully vaccinated US adults	Individuals were selected and offered various incentives, including a paid day off work and monetary rewards of USD 10, USD 100, and USD 1000, to encourage them to receive a COVID booster.	Incentives did enhance the desire to receive the booster; however, the ranking of the incentives indicated that not all monetary rewards were equally impactful.
Sprengholz et al., 2022 [19]	Different Intervention for COVID-19 Primary and Booster Vaccination? Effects of Psychological Factors and Health Policies on Vaccine Uptake	To assess various methods in increasing the COVID-19 vaccine uptake	2701 German participants	An online survey was conducted, in which the first part featured mandates and the second featured incentives for vaccination.	There is a low probability that monetary incentives convince individuals to receive the vaccine, but they may be effective in encouraging booster shots.
Stamm et al., 2022 [20]	Coronavirus vaccine hesitancy among unvaccinated Austrians: Assessing underlying motivations and the effectiveness of interventions based on a cross-sectional survey with two embedded conjoint experiments	To assess underlying motivations’ and incentives’ efficacy in receiving the COVID-19 vaccine	1543 unvaccinated Austrian residents	Participants were offered incentives such as free vaccinations, entry into a vaccination lottery, and an EUR 100 cash reward.	Financial compensation had a greater impact than a vaccination lottery.
Taber et al., 2023 [21]	Experimental Tests of Hypothetical Lottery Incentives on Unvaccinated Adults’ COVID-19 Vaccination Intentions	To evaluate the impact of lottery cash rewards on individuals with no COVID-19 vaccine history	863 US adults	An online survey experiment was conducted in which participants rated their willingness and intention to be vaccinated.	Intentions and willingness to be vaccinated did not significantly vary across the different conditions.

## Data Availability

Not applicable.
